# Community health workers for pandemic response: a rapid evidence synthesis

**DOI:** 10.1136/bmjgh-2020-002769

**Published:** 2020-06-10

**Authors:** Soumyadeep Bhaumik, Sandeep Moola, Jyoti Tyagi, Devaki Nambiar, Misimi Kakoti

**Affiliations:** 1 The George Institute for Global Health, Vishakhapatnam, India; 2 The George Institute for Global Health, Faculty of Medicine, University of New South Wales, Sydney, New South Wales, Australia; 3 Prasanna School of Public Health, Manipal Academy of Higher Education, Manipal, India

**Keywords:** health services research, health systems, infections, diseases, disorders, injuries, systematic review

## Abstract

**Introduction:**

Coronavirus disease (COVID-19), affects 213 countries or territories globally. We received a request from National Health Systems Resource Centre, a public agency in India, to conduct rapid evidence synthesis (RES) on community health workers (CHWs) for COVID-19 prevention and control in 3 days.

**Methods:**

We searched PubMed, websites of ministries (n=3), public agencies (n=6), multilateral institutions (n=3), COVID-19 resource aggregators (n=5) and preprints (n=1) (without language restrictions) for articles on CHWs in pandemics. Two reviewers screened the records independently with a third reviewer resolving disagreements. One reviewer extracted data with another reviewer cross-checking it. A framework on CHW performance in primary healthcare not specific to pandemic was used to guide data extraction and narrative analysis.

**Results:**

We retrieved 211 records and finally included 36 articles. Most of the evidence was from low-and middle-income countries with well-established CHW programmes. Evidence from CHW programmes initiated during pandemics and for CHW involvement in pandemic response in high-income countries was scant. CHW roles and tasks change substantially during pandemics. Clear guidance, training for changed roles and definition of what constitutes essential activities (ie, those that must to be sustained) is required. Most common additional activities during pandemics were community awareness, engagement and sensitisation (including for countering stigma) and contact tracing. CHWs were reported to be involved in all aspects of contact tracing - this was reported to affect routine service delivery. CHWs have often been stigmatised or been socially ostracised during pandemics. Providing PPE, housing allowance, equal training opportunities, transportation allowance, improving salaries (paid on time and for a broad range of services) and awards in high-profile public events contributed to better recruitment and retention. We also created inventories of resources with guiding notes on guidelines for health workers (n=24), self-isolation in the community (n=10) and information, education and counselling materials on COVID-19 (n=16).

**Conclusions:**

CHWs play a critical role in pandemics. It is important to ensure role clarity, training, supportive supervision, as well as their work satisfaction, health and well-being. More implementation research on CHWs in pandemics is required.

**Key questions**

**What is already known?**

- Many countries with existing community health worker (CHW) programmes are mobilising CHWs to assist with COVID-19 response, while some are considering initiating new CHW programmes.

**What are the new findings?**

- In previous pandemics, CHWs have played several roles including for generating community awareness countering stigma and contact tracing. CHW engagement in contact tracing might hamper routine primary service delivery. Policies, guidance and training for these had to be developed.
- Disruption in supply chain, logistics and supportive supervision for CHWs have been common in pandemic scenarios. CHWs have been at increased risk for contracting disease.
- In the course of pandemics, CHWs have experienced stigmatisation, isolation and socially ostracisation. Improved remuneration, additional incentives, public recognition countering societal stigma, provision of psychosocial support and personal protective equipment were reported to be enablers.

**What do the new findings imply?**

- Roles of CHWs in a pandemic context need to be clearly defined with provision of adequate guidance, training and support. Contact listing and identification should be ideally be done by separate trained cadre while CHWs focus on essential service delivery. Ensuring the health, safety, well-being and support for CHWs is essential.
- There is not much evidence about initialisation of new CHW programmes during pandemics. Considering the complexity of barriers faced even in contexts with well-integrated programmes, ambitious programmes need to be considered with caution.
- There is a need for more research on CHWs in pandemics like COVID-19, especially with a focus on equity, gender and economic evaluation.

## Introduction

The COVID-19 outbreak that originated in Wuhan City, China, in December 2019 now affects 213 countries or territories across the globe. Better health workforce utilisation and support are key pillars to enhance health systems capacity.^1 2^ Community health workers (CHWs) constitute a significant proportion of frontline health workforce in many countries^3^ and can potentially play an important role in control and prevention of pandemics like COVID-19.

We received a request to examine the evidence to inform the potential role, enablers and barriers for CHWs during COVID-19 prevention and control from the National Health Systems Resource Centre (NHSRC), India. The NHSRC is a public agency with the mandate to provide technical assistance to India’s National Health Mission, a flagship health systems reform exercise initiated in 2005. An inventory of resources that government decision makers and technical agencies could rapidly scan to help develop guidelines, standards of procedures, advisory notes and communication materials was also deemed to be a useful addendum by the requesters. We conducted a rapid synthesis of global evidence that aimed to:

- Understand key roles, issues, barriers and enablers for CHWs for pandemic response.
- Develop an inventory of resources that could be used to develop guidance, training manuals and information, education and communication (IEC) materials related to COVID-19 for CHWs.

Rapid evidence synthesis (RES) is an emerging arena within the domain of evidence synthesis wherein a balance is struck between accuracy and timelines in the conduct of evidence synthesis to inform health policy and systems decision making.^4^

## Methods

### Approach for the study

Considering the urgency of evidence to inform decision making, we were provided a three-calendar day deadline by the requester. Based on an initial scoping, discussion with the requesters and the emergent nature of COVID-19, we expanded the scope to understand what can be learnt from recent pandemics, that is, severe acute respiratory syndrome (SARS), swine flue, Ebola virus disease (EVD) and Middle East respiratory syndrome coronavirus (MERS-CoV). We followed an RES approach, wherein processes and methods of the traditional systematic review approach were tailored to ensure timeliness.^4^ We took a broad scoping review approach for the RES to support the wide nature of decisions under consideration. While the request form NHSRC was in the context of government-run CHW programmes in India (Accredited Social Health Activists or ASHAs and Aganwadi workers), the scope of the synthesis was not restricted based on governance, financing or scale of the programme, owing to the need to capture the greatest range of relevant evidence in pandemic contexts. There are no reporting guidelines specifically for RES, so we used the Preferred Reporting Items for Systematic Reviews and Meta-Analyses (PRISMA) checklist for scoping reviews.^5^ The PRISMA checklist is presented in online supplementary appendix 1.

10.1136/bmjgh-2020-002769.supp1 Supplementary data

### Eligibility criteria

We included studies that met the following criteria:

- Concept and context: studies or guidance related to roles, issues, challenges and enablers of CHWs during any phase of recent pandemics. The pandemics considered were SARS, swine flu, EVD, MERS-CoV and COVID-19.
- Types of participants: we did not use any specific definition of CHW recognising the fact that there are identified differently in different health systems across the world. We included studies with CHWs as participants (as defined by primary study authors). Synonyms for CHWs including but not limited to the following were also included:
  - Community health aide/practitioner/provider.
  - Lay health worker/aide/practitioner/provider/advisor/promoter.
  - Frontline health worker or community worker.
  - Rural/village/outreach health worker.
  - Community volunteer/worker/surveillance volunteer.
  - Multipurpose health worker or health extension worker or health auxiliary.
- Types of evidence sources: We included following types of evidence sources: - primary research studies (of any design—qualitative or quantitative), any evidence synthesis, guidelines, training materials, advisories, standards of procedures and technical reports.

There was no language, date or geographical restrictions.

### Search methods for identification of records

We searched PubMed given the need to complete the RES in 3 days (search strategy in online supplementary appendix 2). We screened the reference list of all included studies to identify additional records and hand searched (MK and JT) 18 websites of different government ministries (India, Australia and Singapore), public health agencies (from China, US, South Africa, UK, Hong Kong and Australia), multinational agencies (WHO, European CDC and African CDC), COVID-19 resource aggregators available at the time of review (Wiley, Elsevier, Oxford University Press, *New England Journal of Medicine*, *Journal of the American Medical Association*) and preprints (medRxiv) (online supplementary appendix 3).

### Screening, data collection and analysis

Two reviewers independently screened the titles and abstracts of studies for inclusion with a third author resolving consensus (SB, SM and JT). At the full-text level, decision for inclusion or exclusion was done with the consensus of three authors (SB, SM and JT). Data from included studies were extracted using a predefined template by one reviewer (SM, JT, SB and DN) and cross-verified by at least one other reviewer. Cross-verification was not possible for the two French studies as only one of the reviewers knew the language (DN). No assessment of methodological quality of the studies was conducted as a part of methodological tailoring to expedite review process.^4^

We adapted an existing conceptual framework for assessment of CHW performance in primary healthcare^6^ to guide data extraction and narrative synthesis. The framework looks at inputs, programmatic processes, CHW-level and community-level outputs to improve health outcomes in the context of economic evaluation and equity, gender and accountability. The framework is not specific to pandemic response; for ease of reading and clarity, some components of the framework were merged, and issues related to multiple aspects of the framework were mentioned without repetition.

### Patient and public involvement

The RES did not include patients, CHWs or the public in its conduct. The RES has been disseminated to relevant stakeholders and the public as noted in the subsequent section on policy impact and stakeholder engagement.

## Results

### Search results and study selection

**Figure 1:**
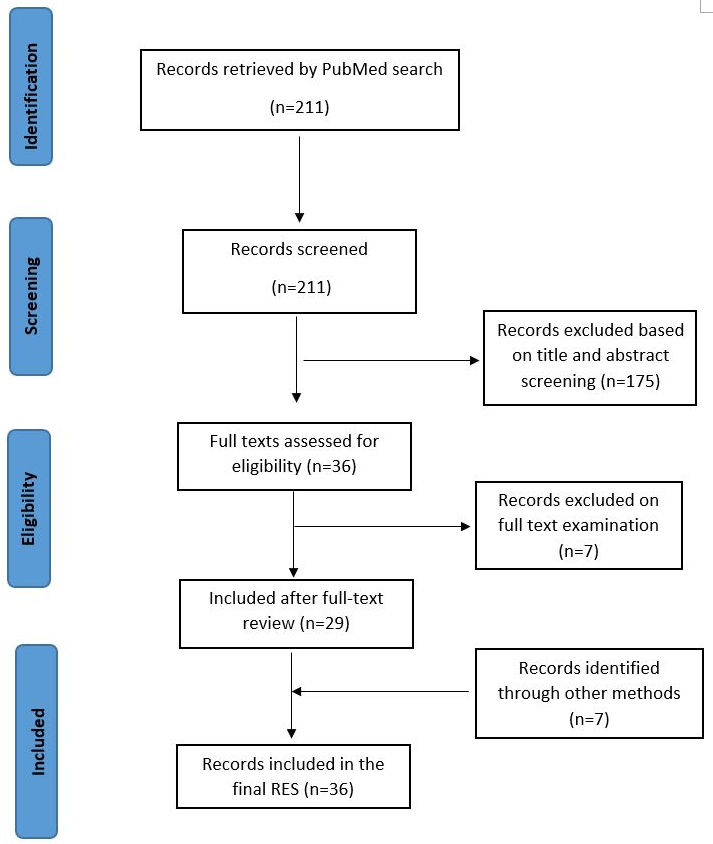
PRISMA flow chart for included studies. PRISMA, Preferred Reporting Items for Systematic Reviews and Meta-Analyses.

We searched PubMed on 21 March 2020 and retrieved 211 records. Following title and abstract screening, full-text articles were retrieved for 36 potentially relevant studies. Seven records were excluded following full-text examination, leaving 29 studies for inclusion. An additional seven studies were identified by screening reference lists of the initial 29 studies that were included. Overall, 36 studies were included in the rapid review. A PRISMA flow chart for the same is presented in figure 1. A list of studies excluded at full-text level is presented in online supplementary appendix 4.

### Characteristics of included studies

A majority of the studies included in the review were qualitative (n=11)^7–17^ followed by descriptive cross-sectional studies (n=9),^18–26^ pretest and post-test studies (n=6),^27–32^ literature reviews (n=3),^33–35^ mixed-methods studies (n=2),^36 37^ guidelines (n=2),^38 39^ one systematic review,^40^ one randomised controlled trial^41^ and one case report.^42^

Key characteristics of the included studies are presented in table 1. Studies were conducted in low-income and middle-income countries or LMICs (Benin, Burundi, Cambodia, Democratic Republic of Congo, Ghana, Guinea, Kenya, Liberia, Mali, Nepal, Nigeria, Sierra Leone, Tanzania, Uganda, Vietnam, Zambia and Zimbabwe) and also in high-income countries (Iceland, UK, USA). The two guidelines were published by the WHO. The three literature reviews and the systematic review had included studies from multiple LMICs in Southeast Asia, South America and Africa.

**Table 1:** Characteristics of included studies and guidelines

Citation	Name of pandemic	Country and setting	Study design and size	Context of CHW involvement	Summary of CHW description (role, training and education, socioeconomic status and remuneration)
**Low-income and middle-income countries**					
Attinsounon *et al*^18^	VHF (Lassa and Ebola), 6 months after end of epidemic.	North Benin (Donga department: Djougou, Ouaké, and Copargo towns).	Cross-sectional survey; 58 volunteers.	Government-run programme for community management of childhood illness has been established in 2009 (PCIME-Communautaire). Community volunteers/community relays (called *relais communautaires*) identified by their peers and in charge of health of children.	CHWs received a half-day training on viral haemorrhagic. These trainings were conducted by health workers from their respective health areas. The study aimed to understand knowledge and attitudes of the CHWs. The mean age of CHWs was 38.7 years; 77% male and 23% female and 50% had primary education, while another 50% had secondary education. Primary occupation of CHWs was 60.3% cultivators, 22.4% resellers and 17.3% craftsmen. CHWs are trained in the home treatment of coughs, diarrhoea and malaria in children under 5 years of age and in the promotion of health in their communities. They also conduct rapid diagnostic tests of malaria.
Bolkan *et al*^26^	Ebola – during pandemic.	Sierra Leone.	Retrospective study; 21 CHWs acted as data collectors.	Study was done as part of a new surveillance initiative to monitor effects of the Ebola epidemic on health services The initiative was a collaboration between the Ministry of Health and Sanitation in Sierra Leone, Karolinska Institute in Sweden, Norwegian University of Science and Technology and the non-governmental organisation CapaCare. Community health officers enrolled in a surgical task-shifting programme that existed previously were involved in the study. This is a collaborative initiative with similar arrangement.	CHWs used as data collectors in retrospective survey to understand indirect effects on health facility function during the pandemic. CHWs were trained in data collection for 2 days and received a tablet with SIM card to allow direct data entering. No other details were provided.
Elston *et al*^36^	EVD. During outbreak period.	Sierra Leone. Rural areas.	Mixed-methods study; 60 IDIs, 6 FGDs; survey in 15 health facilities.	The study was done to measure the impact of the outbreak on population health and health systems with support for the government and through a government run-programme.	HCWs, community workers and social mobilisers were responsible for immunisation and outreach services. The CHWs were recruited for Ebola-related activities and were not paid for this. CHWs also provided community TB DOTS services. CHWs were voluntary workforce. District Health Management Teams supervised the HCWs. HCWs were trained in Ebola treatment.
Farrell et al^7^	Avian influenza (H5N1).	Vietnam, Quang Tri province.	Qualitative study: FGDs and IDIs; 62 village heath workers (both animal and human).	The Avian and Pandemic Influenza Initiative is a community-based surveillance programme that was initiated and funded by the United States Agency for International Development and run by the government. The programme was implemented in a total of 26 communes with 150 villages.	Village animal health worker (VAHWs) and Village human health worker (VHHWs) performed: Targeted community surveillance activities such as regular household visits, communicating with animal and human drug suppliers, liaising with poultry and feed sellers at marketplaces, village education and training, follow-up of rumours and poultry vaccination campaigns. IEC activities related to case definition, reporting and response to animal and human drug suppliers; vaccination schedule and coverage with poultry and feed sellers at marketplaces; regarding correct hand washing and environmental decontamination skills to communities through village events such as farmers’ and women’s union meetings. VAHWs and VHHWs were selected by commune authorities and received a 2–3 day training. Furthermore, ongoing training sessions were held every 6–12 months. Does not specify any other details but its study reports a theme that VAHWs and VHHWs were happy to contribute to community welfare noting that the ‘workload is really big… allowance and salary is very low… there is the risk of disease transmission’ and that the ‘occupation is mainly based on love’.
Vandi *et al*^22^	EVD before, during and after the outbreak.	Sierra Leone, rural Kenema District.	Retrospective cross-sectional study using programme data; 947 (preoutbreak) to 1093 (postoutbreak).	CHW programme is designed and funded by Ministry of Health and Sanitation, Sierra Leone, and is responsible for the recruitment of the CHWs. CHWs are adult volunteers, with the majority literate in English. They are provided a 10-day training course that covers specific clinical duties including symptom-directed screening for malaria, including performing RDTs for malaria; eliciting a history from caregivers; performing basic examinations, such as recording respiratory count; and providing non-injectable treatment for malaria, diarrhoea and pneumonia. CHWs are supervised by PHU staff.	CHW activities to support outbreak management was part of the national response and included social mobilisation, contact tracing and community-based surveillance for EVD. Midoutbreak, CHWs were asked refrain from conducting finger-prick blood tests for rapid malaria diagnostics and basic physical examinations and presumptive treatment was started. CHWs follow a performance-based remuneration system, and while exact details are not provided, it is mentioned that remuneration returned to preoutbreak stipends (equivalent of US$2/month) for transport refund suggesting that top-ups were provided.
Gray *et al*^8^	EVD, early phase and during the outbreak.	Sierra Leone. Urban and rural locations of two districts.	Qualitative study: FGDs and IDI; 16 health workers.	The study was conducted by Médecins Sans Frontières, Ministry of Health and Sanitation to understand the role community interactions with the Ebola response to inform future intervention strategies including issues faced by CHWs. No further details are provided.	CHW roles similar to Vandi *et al.*
Mc Kenna *et al*^29^	EVD, phase not reported.	Sierra Leone, Bo district.	Preinterventional and postinterventional study; 125 CHWs.	Context expected to be similar to Vandi *et al.* Mobile Training and Support programme was funded by Ministry to provide refresher trainings on vaccinations and disease surveillance to CHWs in remote locations and with a special focus on EVD.	CHW roles similar to Vandi *et al*. An interactive voice response technology was used to deliver audio-based refresher trainings to supplement classroom training and the study aimed to assess the training modality. The CHWs were trained by voice recorded content in local languages on the topics of vaccines and (Ebola) disease surveillance and outbreak response. The basis of all training content was the Ministry curriculum for CHWs.
McMahon *et al*^10^	EVD, during peak of outbreak.	Sierra Leone, Bo and Kenema Districts.	Qualitative study: IDIs; 35 health workers.	Context expected to be similar to Vandi *et al.* The frontline providers worked in primary healthcare settings during the recent Ebola outbreak in Sierra Leone. These providers received relatively little attention compared with those working in Ebola treatment units or Ebola patients and their families despite their critical role in the epidemic response.	CHW roles similar to Vandi *et al.* Frontline providers working in PHU setting including community health officers (CHOs), nurses, maternal child health aides, CHWs and laboratory technicians. They were responsible for assisting lactating mothers, visiting sick, dying or grieving family members. There were lower grade health workers (para health professionals), such as porters.
Miller *et al*^37^	EVD, post-outbreak.	Guinea, Liberia and Sierra Leone.	A mixed methods study using IDIs, FGDs and analysis of routine programme data; 102 participants (38 in Guinea, 29 in Liberia and 35 in Sierra Leone.	CHWs were involved in community-based maternal, new born, and child health, CHW services in all the three countries and through a government run programme.	CHWs, traditional birth attendants (TBAs), community health committee (CHC) and traditional healers (TH) were study participants in addition to others. They were responsible for community-based MNCH services, Ebola-related activities and so on. They received limited support in terms of financial incentive; many CHWs had not been supplied with basic equipment such as rain gear and ID cards. CHWs had a supervisory role over TBAs while CHCs made up of community leaders, religious leaders, women’s leaders and youth leaders supervised both CHWs and TBAs. CHWs in these countries worked as volunteers and received small travel allowances and some non-financial incentives, such as boots, rain gear and flashlights. CHWs were trained on to provide Integrated community case management (iCCM) and screen for malnutrition, ‘no touch’ policy, how to care for somebody with EVD in the community and were deployed as ‘active case finders’ during pandemic. TBAs reported that they were given neither training nor infection prevention and control (IPC) materials.
Sidibé *et al*^19^	EVD, during outbreak.	Guinea, four highly affected (Macenta, Conakry, Guéckédou and Kérouané) and less affected (Labé, Fria, Gaoual and Mandiana) districts.	Cross-sectional survey; 120 health facilities. No further details available.	The study was conducted to compare the knowledge, attitudes and practices of routine healthcare providers on suspected EVD cases Government-run programme.	CHWs (also known as prestataires de soins de sant) are posted at health centres (five per centre) and hold responsibility for primary healthcare and prevention activities. They were instructed to follow the ‘Contactless’ policy and avoid the use of needles wherever infection control measures were weak.
Siekmans *et al*^25^	EVD, post-outbreak.	Liberia.	A observational study design used mixed methods; 60 CHWs.	CHWs programme were run by government and the study documents role of CHWs is documented short-term or long-term crises management during EVD. CHWs in general were responsible for referral and community-based treatment of child diarrhoea, malaria, pneumonia, and immunisation Integrated community case management (iCCM) of child illnesses was in function form 2010 to guide CHW work	CHWS conducted house-to-house visits, community meetings, do active case finding or contact tracing. County health officers and health facility staff were responsible for supervising CHWs. CHWs were trained on infection prevention and ‘No Touch iCCM’ as part of training on treatment of child malaria during outbreak.
Stehling-Ariza *et al*^32^	EVD, during outbreak.	Sierra Leone, Kono district.	Pretest and post-test study; not reported.	Context expected to be similar to Vandi *et al.*	Disease surveillance officers, CHWs observed the residents for signs of illness, contact tracing, active surveillance and health education. They were trained on health education to educate residents on symptoms of Ebola.
Wurie *et al*^16^	EVD. Pre-outbreak to during outbreak.	Sierra Leone. Rural areas.	Qualitative study – IDIs; 25 health workers.	Context expected to be similar to Vandi *et al*, and the study was part of a research to understand postconflict health systems.	Community Health Officers (CHOs), nurse health assistants, community health assistant and community health nurse were included. CHWs were involved in outreach and referral services. Additional financial incentives like remote area allowance, performance-based financing payments or accommodation allowances were not received prepandemic. Often had to use own money to buy medicines. CHWs mentioned not receiving regular in-service training.
Wurie *et al*^16^	EVD. Post-outbreak.	Sierra Leone. Hard to reach areas, and urban and rural health facilities.	Qualitative study – IDIs; 23 health workers.	CHW programme in Sierra Leone is largely government managed through public health system. Context expected to be similar to Vandi *et al.*	CHWs and CHOs. Often they left government health facilities to work for non-profits for lack of career progression. Low remuneration of health workers was reported leading to government increasing salaries; however, it was thought to be not commensurate with increased living costs. Additional financial incentives, such as the remote area allowance was provided.
Witter *et al*^17^	EVD. Post-outbreak.	Sierra Leone, Northern Uganda, Zimbabwe and Cambodia. Urban and rural environments.	Qualitative study – IDIs; Cambodia: 19 health workers; Sierra Leone: 23; Uganda: 26; Zimbabwe: 35.	Study was conducted under the realms of a research programme to understand health systems postconflict scenarios.	CHWs, CHOs and nurse aid were included. CHWS often had or loss of pay or it was severely delayed. CHWs were initially provided ration food portions as remuneration. Later payments in rice were converted to cash. CHWS often had to use own resources or borrow or pass costs to patients. Workshops and training on rebuilding relationships with communities was provided.
Otu *et al*^30^	EVD – presurvey was done during epidemic and postevaluation done after epidemic free.	Ondo State, Nigeria.	Prestudy and poststudy design; 94 out of total 203 participants were CHWs (46.3%).	‘Front Line health worker Education and disease Management (FLEM) project’, developed by Instrat and Anadach (non-government) and implemented on the Vecna Cares CliniPAK system under a Qualcomm Wireless Reach grant. They funded an educational intervention to train frontline health workers on mHealth for improving health system functions during the Ebola epidemic.	CHOs, nurses/midwives, community health extension workers responsible for maternal and child health (MCH) care were involved. Participants were trained to use tablet computers and the CliniPAK electronic application.
Patel *et al*^31^	EVD – in midst of outbreak.	Enugu State, Nigeria.	Prestudy and poststudy design (immediately after training); 59 voluntary health advisors (VHAs).	The Healthy Beginning Initiative (HBI) though funded through the government is non-profit run (churches and church-based community networks). HBI promotes individual testing for diseases such as HIV, malaria, sickle cell, hepatitis and syphilis and provides education about prevention of infectious diseases (HIV, hepatitis, syphilis and malaria) through VHAs.	VHAs are lay volunteers at church-based community health programme but is run by government. VHAs were provided an Ebola awareness training session along with training for strategies on dissemination. 61% of VHAs had college education and those with tertiary education had significantly higher knowledge.
Englert *et al*^13^	EVD. Post-outbreak	Uganda. Rural areas.	Qualitative study; n=4.	Uganda had four EVD and five Marburg virus outbreaks from 2000 to 2012 and has significantly high health worker mortality. Uganda has CHW programme as a part of primary healthcare system.	The study included multitude of professionals including nurse aides/nurse assistants and health educators working in government set-up. Most health workers initially fled but later returned after hazard pay was increased. However, they complained this compensation failed to cover the basic necessities.
Hemingway-Foday *et al*^21^	EVD. Late phase, post-outbreak.	Democratic Republic of Congo (DRC). Rural area that is heavily forested.	Descriptive surveillance study; 98 CHWs.	The surveillance team for EVD was under the government, and there was an existing CHW programme.	CHWs (health facility-based workers) were volunteers who supported case finding, case notification and contact monitoring/tracing activities. CHWs supported active case finding, facility-based health workers were trained on the outbreak-specific definitions for suspected, probable and confirmed cases. Participatory methods were used for training and direct supervision from local health authorities.
Plucinski *et al*^23^	EVD during outbreak.	Guinea.	Cross-sectional survey; 219 CHWs.	CHWs who worked for public health centres and were engaged in malaria case management using WHO guidelines, community mobilisation and referral of fever cases to health facility at district level.	CHWs became inactive or left their unpaid malaria case management duties to be paid EVD contact tracers.
Raven *et al*^15^	Ebola 3-5 months after outbreak	Nepal and Sierra Leone.	Qualitative IDIs; 14 health workers in Nepal 25 frontline health workers in Sierra Leone.	National health workers that included health assistants, who were working in Ebola treatment centres and in other government facilities.	Additional risk allowance of approximately US$70 was given to CHOs working in treatment centres and community care centres and all members of the burial team, Additional risk allowance of approximately US$13 were given to contact tracers. Additional support in the form of tents and medicines was given by some agencies to WHO, but this was often delayed by at least 1 week.
Reques *et al*^24^	EVD. Phase two of Ebola’s response.	Guinea. Two rural prefectures.	Descriptive cross-sectional study; 24 supervisors, 22 community workers and 442 contacts.	Community workers and supervisors, as part of the WHO Ebola National Coordination team engaged in contact tracing. This was government run initiative.	At the time of the study, none of the community workers had received their salary, and only 50% and 23% of the households had received the nutrition assistance from World Food Program in Ratoma and Boké, respectively. Contact tracing activities in Guinea involved contact tracing teams, composed of community workers and supervisors. Community workers were recruited among literate persons living in the community and were trained as contact tracers. The community workers were monitored and supervised by physicians and/or epidemiologists. Several difficulties were reported hindering the adequate follow-up of contacts, which highlighted the need to ensure the quality of all aspects of the contact tracing process. An audit tool was used to asses contact tracing activities of the contact tracing team and identifying strengths and weaknesses of their activity.
Ameme *et al*^27^	EVD. Phase not reported clearly.	Ghana.	Pretest and post-test evaluation study; 32 health workers.	A short course for frontline health workers was organised by the Ghana Field Epidemiology and Laboratory Program in the Greater Accra region of Ghana in order to augment their surveillance and outbreak response capacity.	The study had included a small number of CHWs as well as others from the Ghana Health Service and Veterinary Services Department of the Ministry of Agriculture. A 2-week didactic course was conducted with a 10-week field placement evaluation of the course was done by assessment of participants’ outputs during the training as well as pretest and post-test methods. It was a competency-based training workshop on public health surveillance and disease outbreak investigation and response with a focus on EVD. The training was modelled on the Field Epidemiology and Laboratory Training Program short courses for public health workers, which are run globally.
de Vries *et al*^12^	EVD, initial phase.	Uganda. Rural areas.	Qualitative study: FGDs and IDIs; 82.	The study sought to identify the problems encountered with CHW for social mobilisation strategies for early detection and outbreak control during the initial stages of the epidemic. International Federation of Red Cross and Red Crescent Societies funded and organised social mobilisation and psychosocial support during EVD and trained and recruited CHWs.	Local volunteers and local CHWs (VHTs) were recruited (at sub-district level) and trained to provide psychosocial support and built community trust and confidence. Community members have a key role to play in early detection of Viral Haemorrhagic Fevers (VHFs) such as Ebola. They are responsible for the social mobilisation strategies.
Delamou *et al*^20^	EVD. Post-outbreak	Guinea. Public health facilities within 10 rural health districts.	Cross-sectional survey; 299 CHWs, nurses/health technicians: 145, midwives: 70, managers: 48 and physicians: 36.	CHWs were employed in government run health centres/posts and from district and regional level maternities.	They were engaged in providing maternal and child healthcare services (care providers and managers) and in managing febrile cases. CHWs received trained in use of PPE (87% received training) and infection prevention (82% received training) during the EVD outbreak.
Dickmann *et al*^28^	EVD. Initial and late phase of the outbreak;	Uganda. Training was attended by participants from Uganda, Burundi, Zambia, Mali, DRC, Kenya, Zimbabwe, Ghana and Tanzania.	Pretest and post-test study with 100 trainers.	Intensified Preparedness Programme (IPP) was set up in 2015 through government support to work alongside other initiatives by the UN, International Federation of the Red Cross, UNICEF and WHO. IPP was funded by The Rockefeller Foundation and managed by Connecting Organizations for Regional Disease Surveillance. IPP intended to bring together all community-based health ‘shapers’ who had experience in working in EVD outbreaks.	Community-based ‘health shapers’ is a mix of health professionals, community leaders, religious leaders and traditional healers who influenced the narrative of infectious disease management, risk communication and community outreach in their local and national environments. A series of 2-day training programmes to be held in different countries was designed for these influencers. Training programmes on infection control, community engagement, appropriate communication, capacity building, care coordination and culture sensitisation.
Bower *et al*^42^	EVD (phase not reported).	Sierra Leone. Rural community health centre.	Case report.	The study was conducted to understand about possible transmission routes for the woman’s infection by Médecins Sans Frontières. Context similar to Vandi *et al* in terms.	Village traditional birth attendant (VTBA). Not clear if the VTBA was trained and no other specific information provided.
Boyce and Katz^34^	EVD and Zika virus disease (Zika fever).	Countries and setting not reported clearly.	Literature review; not clearly reported.	The context of the narrative review is broad. Broadly, it referred to some Central African countries (eg, Guinea, Liberia) that have pre-existing CHW programmes supported by ministries.	CHWs— were mostly volunteers. They were used to improve community health initiatives, social mobilisation, disease surveillance to manage the risk of infectious diseases (eg, malaria, pneumonia, and tuberculosis), distribution of culturally appropriate health information and supplies. CHWs also acted as a community-level triage system—treating those with minor illness and referring those with more serious disease. This helped decrease the health systems burden. CHWs were trained to assist in the communication or provision of health services and for extending health services at subnational levels in the context of health interventions to carry out defined functions related to healthcare delivery but rarely have formal professional or para professional certifications, or degreed tertiary education.
Armstrong-Mensah and Ndiaye^33^	EVD. Early and late phase but lacks clear details.	South East Asia (Bangladesh, Cambodia, Laos, Pakistan, Thailand and Vietnam), Nicaragua, Chad, Sub-Saharan Africa (Ethiopia, Mozambique, Southern Sudan and Uganda), Latin America (Brazil and Haiti). Community setting.	Literature review.	Global Health Security Agenda (GHSA) is a multilateral and multisectoral initiative that seeks to build and enhance global capacity to prevent, detect and rapidly respond to infectious disease threats by investing in infrastructure, equipment and skilled health workforce personnel. The article advocates for community engagement in GHSA implementation. Most studies included are from contexts of government run CHW programmes (although these were not described specifically).	Several types of CHWs were discussed. These are village health volunteers, community health agents, community health volunteers, village health workers and lay health visitors. Community and religious leaders were trained as social mobilisers in some studies. CHW roles were on the lines of conduct regular monitoring of communicable disease trends and provide regular reporting of pregnancies, births and deaths in rural areas or those with respect to TB and malaria. Majority of CHWs were trained to provide basic health services without receiving any formal professional or paraprofessional certificate or degreed tertiary education.
Perry *et al*^35^	EVD – post outbreak.	West Africa (Ethiopia, Guinea, Liberia and Sierra Leone).	Review/commentary.	The review article was written with the view of noting the role of CHW programmes as a strategy for improving global health security, preventing future catastrophic infectious disease outbreaks and for strengthening health systems.	CHWs provide health education, gather information and deliver basic curative and preventive services at the community and household levels. In some contexts, CHWs were engaged in house to house for community sensitisation, promotion of epidemiologically and culturally appropriate protective practices, data collection and searching for active cases and contacts for EVD. In majority of the cases and in many countries, there was no formal training or education for these CHWs. In one case, dual cadre of CHWs was used consisting of health extension workers (who are professionalised with 1 year of formal training and are paid for serving 2500 people) and health development army volunteers (trained informally by health extension workers and serving 5–10 families).
Selvaraj *et al*^40^	EVD. Phase not reported clearly.	Angola, DRC, Guinea, Liberia, Nigeria, Sierra Leone, Sudan, Uganda, Zaire and Zimbabwe.	Systematic review; 94 studies from 17 different countries.	Outbreaks of deadly infection among health workers are considered red flags that should trigger suspicion for EVD or Marburg virus disease (MVD) and often result in nosocomial spread between staff and patients and then spread back into the community. HWs have been recognised as having an increased risk of infection, owing to their occupational exposure to blood and body fluids, particularly in the absence of appropriate IPC and occupational health and safety measures. Thus, it is important to identify and compare infection and mortality rates and common exposure risks in health workers in EVD and Marburg virus disease (MVD) outbreaks.	CHWs (included traditional healers, mother-and-child aides and contact tracers). Lack of availability of appropriate equipment and/or the lack of training in PPE use during patient care, patient transport and cleaning and environmental disinfection activities. The WHO and International Labour Organization recommend that health workers with EVD and MVD resulting from work activities should have the right to compensation, as well as free rehabilitation and access to curative services.
**High-income countries**					
Nassar *et al*^41^	H1N1 epidemic.	USA. Urban community free-standing birth centre.	Randomised controlled trial 50 pregnant females; daily-automated call group (n=26) and health information group (n=24).	This was not on an existing programme. CHWs implemented an intervention to improve communication between health providers and at-risk patients. The intervention was a communication system consisted of a new automated call monitoring system, applied to second-trimester and third-trimester pregnant women during H1N1 epidemic. This was on the backdrop of CDC recommendation for influenza vaccination to high-risk pregnant women. The study was designed in partnership with CHWs and nurse practitioners/midwives and university scientists.	CHWs were employed full time by the birth centre at the district level. CHWS were trained via a web-based National Insitute of Health (NIH) human subject training (with in-person support). CHWs were engaged in health education session on H1N1 influenza and its dangers for pregnant women, an electronic 8 s thermometer to monitor their temperature when they suspected fever and an educational pregnancy wall calendar to help record appointments and other useful information. CHWs voice was used for the automated call recording for monitoring. Pregnant woman were given unlimited minutes and also a small additional US$35 to pay.
Ives *et al*^14^	Influenza pandemic.	West Midlands region of the UK.	A qualitative study; 5 CHWs.	The study was done to explore perceptions of healthcare workers of UK National Health Services during the influenza pandemic, in order to identify factors that might influence their willingness and ability to work and potential sources of any perceived duty to work.	Community-based HCWs (mostly volunteer) recruited from three NHS Trusts in the West Midlands, one acute teaching, one rural district general and one Primary Care Trust. No special protections were given to staff working in extended roles during the pandemic.
Gunnlaugsson *et al*^9^	EVD, preparedness for pandemic.	Iceland, Landspitali University Hospital.	Qualitative case study – IDI; exact number not reported.	In Iceland, the Directorate of Health highly unlikely that (EVD) undertook an exercise to establish preparedness plans for Ebola in Iceland. The focus of the study is to understand perspectives and experiences of managers and frontline health workers who were involved in the process.	Frontline health professionals worked in the emergency room (ER). This included auxiliary nurses. No other details provided. They attended special training sessions focused on protocols for admission and treatment of a patient with EVD, the donning/doffing of PPE and personal protective measures during patient care.
Guidelines with global context					
WHO^38^	EVD in the midst of the outbreak.	Global.	Guideline report.	During the EVD outbreak in West Africa, contact tracing posed serious challenges, in part as a result of the wide geographical expanse of the EVD outbreak, insufficient resources (human, financial and logistical) and to some extent, limited access to affected communities. It is critical that all potential contacts of suspect, probable and confirmed Ebola cases are systemically identified and put under observation for 21 days. The guidance notes were prepared to articulate and streamline the process of contact tracing with an objective is to facilitate setting up a functional system for conducting systematic contact tracing.	Not applicable.
WHO^56^	COVID-19. During outbreak.	Global. Public healthcare facilities.	Guideline report.	The report is intended for HCWs, healthcare managers and IPC teams at the facility level as they pose an increased risk of infection, due to their occupational exposure to blood and body fluids, particularly in the absence of appropriate IPC and occupational health and safety measures.	Not applicable.

CHW, Community Health Worker; DOTS, Directly observed treatment, short-course; EVD, Ebola virus disease; FGDs, focus group discussions; IDIs, in-depth interviews; IEC, information, education and communication; PHU, peripheral health unit; PPE, personal protective equipment; RDT, Rapid Diagnostic Test; TB, Tuberculosis; VHF, Viral Haemorrhagic Fever.

Reporting for context of CHWs programmes in the studies was not consistent. Notwithstanding this, the bulk of the evidence from LMICs is from countries that had existing government managed CHW programmes (government or aid funded) with a few exceptions. These exceptions were an existing government-funded programme in Nigeria managed by a non-government faith-based organisation and a new government CHW program being initiated in Vietnam.^7^ In addition, another study looked at pandemic related training being provided by non-government agencies to existing CHWs in Nigeria.^30^ CHWs in LMICs are volunteers with no permanent base-pays and are paid small amounts (in cash or kind) on the basis of actual service delivery. The high-income country evidence pertained to pandemic preparedness in Iceland^9^; USA, wherein a new CHW program was initiated during a pandemic wherein CHWs were integrated as staff; and the UK where potential availability of volunteers for CHWs was studied.^14 41^ As such, the evidence from high-income countries is very limited, and unless otherwise stated, evidence in subsequent section pertains to LMIC contexts.

### Summary of findings on role, issues, enablers and barriers for CHWs in pandemic response

The included studies examined the role of CHWs in disease outbreak situations, in addition to exploring the barriers and enablers of working in pandemics. Overall, the studies reported that CHWs had a role to play in terms of community engagement (mostly in rural areas) and creating awareness during pandemic responses. The key findings from the included studies are presented as per the elements of the conceptual framework.^6^

#### Input in CHW programmes during pandemic responses

Myriad inputs and planning were required to ensure effective pandemic response through CHWs. These are summarised in subdomains below:

- Policies related to CHW task/roles. Policies and guidance related to specific roles and tasks for CHWs were reported in many studies.^9 21 30 34 37 38^ These policies largely aimed to distinguish tasks as essential (routine activities that need to be continued but with modifications for decreased transmission risk), non-essential (non-essential activities that could be postponed) and additional activities that need to be carried. New roles and tasks for CHWS pertaining specifically to pandemic control varied immensely. This included community awareness (related to disease risk factors and symptoms, appropriate preventive practices and countering stigma), engagement and sensitisation,^8 12 16 18 21 22 25 28 29 31–37 42^ contact tracing,^21 22 24 25 32^ visiting sick or grieving community members,^10^ targeted community surveillance for prevention using one-health approaches^7^ and data collection to understand the impact of a pandemic^26^ in LMICs. In USA, they were engaged for better risk communication to high-risk groups.^41^ The other two studies from high-income countries did not indicate specific CHW roles.

- The WHO’s guidance on contact tracing recommends contact identification and listing to be conducted by a trained epidemiologist or surveillance officer, while contact follow-up might be done through CHWs.^38^ However, polices to involve CHWs in aspects of contract tracing had been developed and implemented during EVD, but their appropriateness is not well understood.^21 22 24 25^ It has further been noted that CHWs had completely abandoned their routine unpaid malaria case management duties over preferences to the paid contact tracing work.^23^ Changes in policies and standards of procedures for routine service delivery (eg, ‘no-touch’ policies for community level screening and treatment) were also reported.^25 32 37^ Policies for defining CHW roles were reported to be crucial for the purpose of bringing accountability and building trust in health systems, although this was not discussed more specifically.

- Logistics and funding for CHW programmes. Disruptions in drug and equipment supplies were common during pandemics and policies and mechanisms to address them from an early phase of the outbreak were reported to be important.^5^ ^10^ ^16^ In addition, shortfalls in routine supportive supervision methods may be expected. Sustained investments in CHWs were required during pandemic response including revisiting financial investments (discussed subsequently) for optimal outcomes.^34^ Issues in relation to financial incentives for CHWs are further discussed in section on CHW motivation and well-being.
- Governance and stakeholders. A wide range of stakeholders in the community needed to be engaged. The evidence base on stakeholder engagement is mostly from LMICs with existing CHW programmes where they were additionally engaged for COVID-19 activities and has been discussed in a subsequent domain on community support.
- Information management systems for CHWs in pandemics. Operational support using information management systems and digital health technology were reportedly used for supporting and monitoring CHW programmes. Honesty, transparency of communication and reciprocity of information with CHWs were noted to be beneficial.^9 14 21^ Many studies reported that pandemics impacted supervision and support for CHWs and health management information systems. Disease surveillance systems (reliant on passive reporting from health facilities) became almost entirely non-functional in some cases.^36 43^ In Vietnam, a new community based surveillance component was developed, in which CHWs played a critical role.^22^ CHWs recorded their daily activities in registers, and their reports were collated by the peripheral health unit supervisor and entered electronically, which formed the basis of information reaching the Ministry. The requirement to travel long distance to attend supervision meetings, health worker shortage, facility closures, restrictions on movement and increased prioritisation pandemic work were identified as key challenges for routine supervision and health information systems management.^25 37^

Programmatic process and health systems were crucial for effective service delivery by CHWs during pandemics.

#### Supportive systems, development, competency and well-being for CHWs

CHWs and their supervisors required appropriate training and supportive supervision for community sensitisation, awareness and risk communication during pandemic outbreaks, and the lack of these was noted as a significant barrier to effective service delivery.^7 9 13 16–18 20 21 25 29 32 34 37 38 40^

The nature, content and modality of training during pandemic varied immensely depending on context and specific role carved out for CHWs: as such what constitutes appropriate training for CHW might be very context specific. Components of training programmes reported focused on:

- Changes in protocols for existing service delivery pertaining to screening or managements.
- Education and awareness on the pandemic disease condition for CHWs.
- For generating awareness, risk communication and community sensitisation strategies on the pandemic condition to key client groups of CHWs or as in the area they served completely.
- Disease surveillance, contact tracing and outbreak response.
- Transportation and management of suspect or confirmed cases of those affected by the pandemic disease condition.
- Use of personal protective equipment (PPE) and other infection control practices.
- Rebuilding community relationships postoutbreak.

In most of these studies, it was reported that appropriate training in skills and knowledge related to disease outbreaks led to an improvement in awareness of the disease, screening and reporting. In qualitative studies, it was reported that training changed in perceptions of CHWs, leading to more proactive involvement in disease prevention and control. In Sierra Leone, for instance, the Mobile Training and Support programme (an e-health training for CHWs on pandemics) used a decentralised reporting structure with outreach to individual CHWs and was reported to be useful.^29^ Appropriate training helped CHWs overcome fear and become more confident about providing and delivering care and helped them deal with a broad range of challenges. Transparency in communication that included sharing of information from supervisors motivated CHWs to be involved and mitigated the fear frequently encountered during pandemics.

CHWs were reported to be at an increased risk of exposure due to lack of or insufficient or incorrect usage of PPE.^10 18 19 40^ In previous disease outbreaks, training sessions on pandemic preparedness were focused on protocols, and the use of appropriate and adequate PPE, during patient care. There is some evidence that training and availability of PPE resulted in CHWs being more confident to cope with managing the disease outbreak.^10 13 16 17 20 40 42^

During previous pandemics, CHWs have faced stigmatisation, isolation and in some cases were even socially ostracised.^15 33 37^ The studies reported that communities often saw them as ‘carriers for infection’ owing to the proximity (real or perceived) with those affected by the outbreaks as well as high mortality rates among healthcare workers in general. CHWs often isolated themselves to protect their ‘loved ones’ from disease and stigma. As noted in the subsequent section, availability of PPE boosted confidence in CHWs but its effect on community perceptions about CHWs has not been studied.

CHWs used several coping strategies including peer support, finding renewed purpose in continuing to serve the community and turning to religion to guide strength.^15^ None of the studies reported preparedness to mitigate well-being for CHWS, but post facto efforts like peer and family support (praying together before work or through social media platform like WhatsApp) were reported to enhance CHW well-being.^15^ Furtherore, workshops that provided emotional support and ways to deal with the social stigma helped CHWs cope with seeing patients and colleagues dying from the disease. Studies reported that improved working conditions, provision of housing allowance, equal training opportunities, transportation/remote area allowance (or provision of bicycles in rural areas), and improved salaries (paid on time and for a broad range of services), awards in high-profile public events contributed to better recruitment and retention of CHWs during pandemics.^13 15 16 33 34^ Some studies also noted that CHWs often had to bear the cost on their own or even borrow money to provide services or medicines to those they served—acting as an important challenge for motivation.^16 17^

#### Support from community-based groups for community access and community-centred care

Community engagement and sensitisation were reported to address knowledge gaps related to disease outbreak and discourage discrimination and stigmatisation towards CHWs.^15 33^ Coercive laws against community members initiating discrimination and stigmatisation towards CHWs were not reported. Information sharing in the community as well as engagement of community leaders were reported to help build trust in CHWs, leading to better access as well as satisfactory experience in care received.^8 28 33 35 37^ Community elders and village heads should be engaged and involved to rebuild trust, leading to an increased use of services. We did not find any evidence pertaining to aspects of community support and access from high-income countries.

#### Community-level outputs and health outcomes of CHW-led pandemic response

A study noted that the social standing (in terms of social status) of CHWs empowered them in terms of their participation in pandemic responses.^34^ Community health policies for social mobilisation and community engagement strategies were found to build trust and increase utilisation of services, which were found to be useful in pandemic response.^8 15 33 37^ Engaging CHWs in the early phases of outbreaks potentially improved overall response procedures and adaptive resilience.^34^ It was reported that CHW-led programmes led to improved knowledge and awareness among farmers leading to improved motivation for reporting of avian influenza.^7^ In another study, it was noted that reporting for malaria by CHWs improved from 59% (preoutbreak) to 95% (during outbreak) and 98% (postoutbreak) owing to ‘sustained investments in CHWs through provision of incentives, supervision and provision of adequate reporting tools’.^22^ In general, training of CHWs led to improved knowledge, service quality and well-being, as has been noted in preceding sections. One study from rural Uganda reported that CHW-delivered psychosocial support to build community trust and confidence was often not available for disadvantaged and poor families owing to lack of accountability measures.^12^ As such, it is important to build adequate governance and reporting structures and use an equity-focused approach in programming.

#### Equity, gender, accountability and economic evaluations

We did not find any economic evaluations related to CHWs in pandemic responses. Issues around equity, gender and accountability were seldom studied, including in relation to the populations to whom CHWs provided their services. Specific equity-focused studies were scarce, but female CHWs had a different set of challenges, which has been discussed in an earlier section pertaining to CHW support and well-being.

### Summary of inventory of resources to guide resource development

We created three inventories to guide and expedite resource development. The first inventory (online supplementary appendix 5) compiled 24 documents. Apart from guidance documents useful for health workers (not just CHWs) during pandemics, the inventory also included resources for ensuring occupational safety and well-being and listing actions for local health departments and subnational planning.

The second inventory included 10 guidelines (online supplementary appendix 6) and advisories on self-isolation practices that have been released by various government health departments to control COVID-19. They provide instructions for the 14-day home quarantine in non-healthcare settings, that is, at home for those with close contact to COVID-19 cases, inbound travellers from affected areas and people sharing the same home with a suspect or positive COVID-19 person.

The third inventory consisted of list of 16 different IEC resources (online supplementary appendix 7). They addressed frequently asked questions, list COVID-19 myths and facts, dos and don’ts for the general public and specific communication messages for high-risk population and individuals showing symptoms available at the time of the request.

## Discussion

### Summary of main results and their implications for policy and practice

#### Role of CHWS during a pandemic

Evidence from our RES pertains mostly to well-established government-run CHW programmes in LMICs. The implications of introducing new CHW programmes in the midst of a pandemic, as is being planned by the UK,^44^ are not well understood, but considerable challenges might be expected considering the larger body of literature around community trust and systems integration being critical for success.^45–48^

In countries with well-established CHW programmes, roles and tasks of CHWS change substantially during pandemics. There is a need to change the normal standards of procedures of conducting routine activities—as for example instituting ‘no-touch policy’. Relevant, tailored and continuously updated guidance, training and supportive supervision should be planned for. Most common additional activities for CHW during pandemics were community awareness, engagement and sensitisation (including for countering stigma) and contact tracing. However, when CHWs were involved in contact tracing, substantial reduction in delivery of routine but essential health service delivery took place. This is crucial considering the larger body of evidence from previous pandemics showing consequences on population health owing to disruption in routine service delivery.^43 49 50^ WHO Guidance mandates possible use of CHWs only for contact follow-up component of contact tracing.^38^ The bulk of the contact tracing work involves contact identification and listing, and it should be conducted by a separate trained cadre, as mandated by WHO. Effectiveness of different cadres for conducting contact tracing is beyond the scope of the RES. Yet, our evidence suggests that not involving CHWs in contact tracing may also safeguard essential primary healthcare service delivery, which is a key concern in any pandemic.

#### Key considerations for effectively mobilising CHW in a pandemic

There is a wide variation in CHW programmes in most countries in alignment with how they are defined and what they tasks they are allocated in normal times and in terms of their level of education and training, modality and degree of engagement with health system and remunerations they receive for the same.^51^ As such, in terms of practical decision making, the background context will determine considerations for effective CHW mobilisation during a pandemic. However, clear guidance on changed roles and tasks—vis-à-vis essential activities that need to be sustained (with modification), non-essential tasks that can be postponed and additional pandemic activities that need to be performed—is critical. As noted in our RES, disruption in supply chain, logistics and supportive supervision is expected and should be accounted for when guidance and training are being instituted. These apart, supportive structures related to governance, sustained financing and health information systems need to be carefully planned with built in strategies for course correction and updating in light of new information and developments. All these imply the need for training to be carefully planned and anticipating potential scenarios and consequent mitigation plans.

During previous pandemics, CHWs experienced stigmatisation, isolation and were socially ostracised, and the same might be expected during COVID-19 without appropriate investment and support the same. Governments should thus make provisions for psychosocial support in the form of peer support and professional help. While the RES does not provide any particular information pertaining to proactive approach to prevent stigmatisation of CHWs in the first place, general principles of antistigma programmes^52^ indicated community awareness and engagement can be key. As such engagement for and by CHWs with community leaders to develop culturally appropriate messages might serve the dual purpose of awareness for prevention as well as countering stigma. Futhermore, developing an a priori pandemic communication plan and engaging with CHWs and community leaders that aim to build trust, engage with affected populations, and integrate risk communication into health and emergency response systems would be useful.^53^ Our RES indicated that training and aavailability of PPE resulted in CHWs being more confident to cope with managing the disease outbreak. The effect of PPE availability for CHWs on communities is not reported. However, an effective health system which takes care of local CHWs might potentially build community trust in addition to ensuring CHW safety.

CHWs in most contexts, and as we found in most of the included studies, have a volunteer status without any base salary, long-term security or other employment benefits like retirement benefits, medical or life insurance.^54^ Performance-based incentives (financial or in-kind) leads to CHWs focusing more on tasks that are remunerated. As such, in pandemic, wherein many of the ordinarily remunerated, tasks are suspended and additional tasks are being allocated might be unsettling for CHWs. More broadly, recognition as staff and moving towards a base salary structure in alignment with the 2018 WHO recommendation against a performance-based incentive structure^55^ during a pandemic should be planned. This will not just be beneficial for health systems performance but is also imperative from a rights perspective. There is, however, need for more research on this domain. Provisions for additional transport allowance, accommodation and child support should also be considered owing to the need to protect families of CHWs from infection. Awards and recognition in a high-profile manner might also be useful.

In many countries most, if not all, CHWs are women and are subject to gender norms (domestic work being one example) as well as other gendered vulnerabilities and risks (like domestic violence).^56–58^ Sustained investment together with governance and information systems support for this cadre are important. Furthermore, as argued elsewhere, CHWs are part of a larger ecosystem of health system wherein ensuring trust and accountability is essential, more so during a pandemic.^49^

### Implications for research

We took a broad scoping approach in the RES to understand issues around CHWs in pandemic response. Conduct of research related to health workers during pandemics is extremely challenging and, as such, high-quality evidence from trials may be neither feasible nor ethical. However, the use of cross-sectional surveys, before-and-after studies, time-series studies and qualitative study designs is feasible. Implementation research related to key pandemic response functions like contact tracing, community awareness and antistigma programmes is crucial to understand issues related to human resources for health and better pandemic control. Equity, gender and accountability dimensions together with economic evaluations are major gap in the literature that need to be studied.

### Strength and limitations of the review process

In terms of the strengths of this review, a robust and transparent search strategy was used to identify relevant studies of interest. Broadening the scope to include multiple recent pandemics enabled us to get more meaningful evidence from a broader but relevant literature to inform decision making. We searched only one database, but we had extensively used other methods to identify other research. The identification of seven studies through hand-searching of websites and reference screening demonstrate the robust application and utility of the approach. We have not hand-searched government, ministry and agency websites in China and other South American countries (which have large-scale CHW programmes), but we found reviews that included studies from South American Countries. Being an RES, we acknowledge such limitations owing to the time constraints we faced. CHWs programmes are in essence a complex health systems intervention. Our broad scoping approach implies specific issues like accountability, scaling up, impact of training, education, socioeconomic conditions, supervision and magnitude of remuneration of CHWs on service delivery or effectiveness of alternatives to CHW programmes is not within the scope of the RES.

### Policy impact and stakeholder engagement

We submitted the key policy considerations along with a shorter version of the RES to the requester and released it publicly on our institutional website considering the immediacy of decision making. The RES has been used nationally for the development of training resources, recommendation adoption in Odisha (a state in eastern India) and has been referred to in WHO policy note on violence against women^56^ during COVID-19. The inventories have also been used extensively for preparation of checklists for rural and urban primary health centre preparedness to tackle COVID-19.^59 60^ Our detailed experience in relation to stakeholder engagement for uptake of RES will be captured in a separate paper.

## Conclusion

In conclusion and based on our findings, CHWs play an important role in the prevention and control of pandemics like COVID-19. This is relevant and feasible in countries that already have existing CHW programmes. However, there is a need for role clarity, further training and regulation to ensure preparedness of CHWs in pandemic-like situations. The use of CHWs in countries without pre-existing CHWs programmes may be challenging considering the health systems reorientation needs and the lack of established community relationships. Applicability of available evidence on CHWs for pandemic response should be considered by such countries before embarking on ambitious CHW programmes amid a public health crisis.
