# Supplementary material for: Community health workers for pandemic response: a rapid evidence synthesis
Source: BMJ Glob Health. 2020 Jun 10;5(6):e002769. doi: 10.1136/bmjgh-2020-002769 (PMC7292038; doi:10.1136/bmjgh-2020-002769)
Supplement: Supplementary data [file bmjgh-2020-002769supp001.pdf]

Appendices for Community health workers for pandemic response: a rapid evidence synthesis

Bhaumik S, Moola S, Tyagi J, Nambiar D, Kakoti M

Contents

Appendix 1 : PRISMA-ScR Checklist 2

Appendix 2– Search Strategy for PubMed 4

Appendix 3– List of organisations, and other unpublished resource databases that were hand searched 5

Appendix 4 – Table of studies excluded at full text phase 6

Appendix 5 – Inventory of relevant guidelines on COVID-19/pandemic control and response for health workers 7

Appendix 6 – Inventory of self-isolation guidelines in relation to COVID-19 control 11

Appendix 7 – Inventory of IEC materials in relation to COVID-19 control and response produced by different organisations/authorities worldwide 12

Appendix 1 : PRISMA-ScR Checklist

SECTION	ITEM	PRISMA-ScR CHECKLIST ITEM	REPORTED
**TITLE**			
Title	1	Identify the report as a scoping review.	Yes, as RES
**ABSTRACT**			
Structured summary	2	Provide a structured summary that includes (as applicable): background, objectives, eligibility criteria, sources of evidence, charting methods, results, and conclusions that relate to the review questions and objectives.	Yes
**INTRODUCTION**			
Rationale	3	Describe the rationale for the review in the context of what is already known. Explain why the review questions/objectives lend themselves to a scoping review approach.	Yes
Objectives	4	Provide an explicit statement of the questions and objectives being addressed with reference to their key elements (e.g., population or participants, concepts, and context) or other relevant key elements used to conceptualize the review questions and/or objectives.	Yes
**METHODS**			
Protocol and registration	5	Indicate whether a review protocol exists; state if and where it can be accessed (e.g., a Web address); and if available, provide registration information, including the registration number.	Yes
Eligibility criteria	6	Specify characteristics of the sources of evidence used as eligibility criteria (e.g., years considered, language, and publication status), and provide a rationale.	Yes
Information sources*	7	Describe all information sources in the search (e.g., databases with dates of coverage and contact with authors to identify additional sources), as well as the date the most recent search was executed.	Yes
Search	8	Present the full electronic search strategy for at least 1 database, including any limits used, such that it could be repeated.	Yes
Selection of sources of evidence†	9	State the process for selecting sources of evidence (i.e., screening and eligibility) included in the scoping review.	Yes
Data charting process‡	10	Describe the methods of charting data from the included sources of evidence (e.g., calibrated forms or forms that have been tested by the team before their use, and whether data charting was done independently or in duplicate) and any processes for obtaining and confirming data from investigators.	Yes
Data items	11	List and define all variables for which data were sought and any assumptions and simplifications made.	Yes

SECTION	ITEM	PRISMA-ScR CHECKLIST ITEM	REPORTED
Critical appraisal of individual sources of evidence§	12	If done, provide a rationale for conducting a critical appraisal of included sources of evidence; describe the methods used and how this information was used in any data synthesis (if appropriate).	Yes
Synthesis of results	13	Describe the methods of handling and summarizing the data that were charted.	Yes
**RESULTS**			
Selection of sources of evidence	14	Give numbers of sources of evidence screened, assessed for eligibility, and included in the review, with reasons for exclusions at each stage, ideally using a flow diagram.	Yes
Characteristics of sources of evidence	15	For each source of evidence, present characteristics for which data were charted and provide the citations.	Yes
Critical appraisal within sources of evidence	16	If done, present data on critical appraisal of included sources of evidence (see item 12).	Yes
Results of individual sources of evidence	17	For each included source of evidence, present the relevant data that were charted that relate to the review questions and objectives.	Yes
Synthesis of results	18	Summarize and/or present the charting results as they relate to the review questions and objectives.	Yes
**DISCUSSION**			
Summary of evidence	19	Summarize the main results (including an overview of concepts, themes, and types of evidence available), link to the review questions and objectives, and consider the relevance to key groups.	Yes
Limitations	20	Discuss the limitations of the scoping review process.	Yes
Conclusions	21	Provide a general interpretation of the results with respect to the review questions and objectives, as well as potential implications and/or next steps.	Yes
**FUNDING**			
Funding	22	Describe sources of funding for the included sources of evidence, as well as sources of funding for the scoping review. Describe the role of the funders of the scoping review.	Yes

From: Tricco AC, Lillie E, Zarin W, O'Brien KK, Colquhoun H, Levac D, et al. PRISMA Extension for Scoping Reviews (PRISMAScR): Checklist and Explanation. Ann Intern Med. 2018;169:467–473.

Appendix 2– Search Strategy for PubMed

No.	Search terms	No. of hits
#1	"community health worker*"[tw] OR "lay health worker"[tw] OR "lay health workers"[tw] OR "frontline health workers"[tw] OR "close-to-community providers"[tw] OR "frontline health providers"[tw] OR "CHW"[tw] OR "CHWs"[tw] OR "LHW"[tw] OR "LHWs"[tw] OR "lady health workers"[tw] OR "rural health worker*"[tw] OR "accredited social health activist"[tw] OR "ASHA worker"[tw] OR "ASHA workers"[tw] OR "community surveillance volunteer*"[tw] OR "female multipurpose health worker*"[tw] OR "community case management worker*"[tw] OR "health extension worker*"[tw] OR "outreach worker"[tw] OR "health extension worker*"[tw] OR "community health agent*"[tw] OR "community health aide"[tw] OR "community health aides"[tw] OR "village health worker"[tw] OR "village health workers"[tw] OR "health auxiliary"[tw] OR "health auxiliaries"[tw] OR "barefoot doctor"[tw] OR "community health practitioner"[tw] OR "community health practitioners"[tw] OR "lay health advisor"[tw] OR "lay health advisors"[tw] OR "lay health promoter"[tw] OR "lay health promoters"[tw] OR "community worker"[tw] OR "community workers"[tw]	4551
#2	(("2019-nCoV" OR "2019nCoV" OR "COVID-19" OR "SARS-CoV-2" OR "SARS-2" OR (wuhan AND coronavirus))) OR (("COVID-19"[Supplementary Concept] OR "severe acute respiratory syndrome coronavirus 2"[Supplementary Concept])) OR "Wuhan seafood market pneumonia virus"	1429
#3	Hemorrhagic Fever, Ebola[MH] OR Ebola[tw] OR SARS[tw] OR coronavirus[tw] OR "severe acute respiratory syndrome" [tw] OR "SARS Virus"[MH] OR "SARS Virus"[tw] OR "SARS-CoV"[tw] OR "SARS-Associated Coronavirus"[tw] OR "sudden acute respiratory syndrome"[tw] OR "swine flu"[tw] OR "swine influenza"[tw] OR "HIN1"[tw] OR "Middle East respiratory syndrome coronavirus"[tw] OR "MERS-CoV"[tw] OR pandemic[tw] OR pandemics[tw] OR "disease outbreak"[tw] OR "disease outbreaks"[tw] OR outbreak[tw] OR "outbreaks"[tw] OR epidemic[tw] OR epidemics[tw]	240235
#4	#2 OR #3	240473
#5	#1 AND #4	**211**

Appendix 3– List of organisations, and other unpublished resource databases that were hand searched

**Organisation and Weblink**
1. European CDC: www.ecdc.europa.eu<br>2. Africa CDC www.africacdc.org<br>3. China CDC www.chinacdc.cn<br>4. World health Organization www.who.int<br>5. CDC Atlanta www.cdc.gov<br>6. Ministry of health and family welfare www.mohfw.gov.in<br>7. Ministry of health Singapore www.moh.gov.sg<br>8. National Institute of Communicable Disease www.nicd.ac.za<br>9. Australian Government Department of Health www.health.gov.au<br>10. U.S. Department of Labor Occupational Safety and Health Administration www.osha.gov<br>11. Public health England www.gov.uk<br>12. Centre for health protection. Department of health the Government of the Hong Kong special administration region https://www.chp.gov.hk/files/pdf/ic_advice_for_nid_in_healthcare_setting.pdf<br>13. New England Journal of Medicine https://www.nejm.org/coronavirus<br>14. Wiley Online Library https://novel-coronavirus.onlinelibrary.wiley.com/<br>15. Elsevier https://www.elsevier.com/connect/coronavirus-information-center<br>16. medRxiv and bioRxiv http://connect.medrxiv.org/relate/content/181<br>17. Oxford Academic https://academic.oup.com/journals/pages/coronavirus<br>18. JAMA Network https://jamanetwork.com/journals/jama/pages/coronavirus-alert

Appendix 4 – Table of studies excluded at full text phase

No.	Name of Study	Reason for exclusion
1.	Acceptability of pandemic A(H1N1) influenza vaccination by Essential Community Workers in 2010 Alicante (Spain), perceived seriousness and sources of information	Wrong population
2.	The Impact of the SARS Epidemic on the Utilization of Medical Services: SARS and the Fear of SARS	Wrong population
3.	West African countries focus on post-Ebola recovery plans	Wrong Population
4.	Integrated disease surveillance and response strategy for epidemic prone diseases at the primary health care (PHC) level in Oyo State, Nigeria: what do health care workers know and feel?	It does not include CHWs
5.	Health inequalities and infectious disease epidemics: a challenge for global health security	It does not include CHWs
6.	Lives on the line? Ethics and practicalities of duty of care in pandemics and disasters	It does not include CHWs
7.	Reduced vaccination and the risk of measles and other childhood infections post-Ebola	It does not include CHWs

References:

1. Caballero, P., Tuells, J., Duro-Torrijos, J. L., & Nolasco, A. (2013). Acceptability of pandemic A(H1N1) influenza vaccination by Essential Community Workers in 2010 Alicante (Spain), perceived seriousness and sources of information. Preventive Medicine, 57(5), 725–728. doi:10.1016/j.ypmed.2013.08.008
2. Chang, H.-J., Huang, N., Lee, C.-H., Hsu, Y.-J., Hsieh, C.-J., & Chou, Y.-J. (2004). The Impact of the SARS Epidemic on the Utilization of Medical Services: SARS and the Fear of SARS. American Journal of Public Health, 94(4), 562–564. doi:10.2105/ajph.94.4.562
3. Green, A. West African countries focus on post-Ebola recovery plans. The Lancet, 2016. 388(10059), 2463–2465.
4. Jinadu KA, Adebiyi AO, Sekoni OO, Bamgboye EA. Integrated disease surveillance and response strategy for epidemic prone diseases at the primary health care (PHC) level in Oyo State, Nigeria: what do health care workers know and feel? Pan Afr Med J. 2018 Sep 7;31:19.
5. Quinn SC, Kumar S. Health inequalities and infectious disease epidemics: a challenge for global health security. Biosecur Bioterror. 2014 Sep-Oct;12(5):263-73.
6. Simonds, A. K., & Sokol, D. K. (2009). Lives on the line? Ethics and practicalities of duty of care in pandemics and disasters. European Respiratory Journal, 34(2), 303–309.
7. Takahashi S, Metcalf CJ, Ferrari MJ, Moss WJ, Truelove SA, Tatem AJ, Grenfell BT, Lessler J. Reduced vaccination and the risk of measles and other childhood infections post-Ebola. Science. 2015 Mar 13;347(6227):1240-2.

Appendix 5 – Inventory of relevant guidelines on COVID-19/pandemic control and response for health workers

No.	Document Title	Issuing authority/ Country of origin/Context	Notes
1.	Responding to community spread of COVID-19	WHO, Global	Provides comprehensive guidance on response to community spread of COVID-19. Includes links and references to resources for various other aspects of COVID response
2.	Interim Guidance for Public Health Personnel Evaluating Persons Under Investigation (PUIs) and Asymptomatic Close Contacts of Confirmed Cases at Their Home or Non-Home Residential Settings	CDC, USA	Provides guidance on infection prevention and control practices when risk assessment and public health management of persons with potential COVID-19 is performed at a home or non-home residential settings
3.	Guidance for Healthcare Worker Self-Monitoring and Work Restriction: In the Presence of Sustained Community Transmission of Coronavirus Disease 2019	Michigan Department of Health & Human Services, US	Guidance for health workers on how to self-monitor themselves during community spread of a pandemic
4.	Mental Health and Psychosocial Considerations During COVID-19 Outbreak	WHO, Global	Focusses on mental health of healthcare workers while responding to COVID distress
5.	Pandemic Influenza Preparedness and Response Guidelines for Healthcare Workers and Healthcare Employers	Occupational Safety and Health Administration, USA	Pages 15-16: Infection control precautions for healthcare workers, guide on work practices and use of personal protective equipment
6.	2019 Novel Corona Virus Disease Outbreak: What Healthcare Workers Should Know	Africa CDC, Africa (African Union)	Provides details on precautions a health care worker should take to protect themselves which is generic in nature and may be used as an instruction guide for CHWs
7.	Risk assessment and management of exposure of health care workers in the context of COVID-19	WHO, Global	Aids in risk assessment for HCWs after exposure and provides recommendations for their management. Provides recommendations for health workers at low risk for COVID-19 (Page-6) which might be useful in the context of CHWs.

8.	Public health management of persons, including health care workers having had contact with COVID-19 cases in the European Union	European CDC, Europe	This document provides guidance for EU/EEA public health authorities on the management of persons, including healthcare workers, who had contact with COVID-19 cases. May be applicable for community health workers (low risk exposure: page 2).
9.	Protect Your Patients and Staff from COVID-19: CDC's Recommended Infection Control Procedures[1]	CDC, USA	Recommendation on infection control and protecting patients and staffs. The staff comprises of health workers, but the additional recommendations could be contextual to the CHWs as well.
10.	Implementation of Mitigation Strategies for Communities with Local COVID-19 Transmission	CDC, USA	Pages 8-9: Mitigation strategies and activities according to level of community transmission or impact of COVID-19 by setting
11.	Australian Health Management Plan for Pandemic Influenza	Department of Health, Australia	PPE for health workers, public health officials and other workers which includes CHWs in direct contact with infected (symptomatic) individuals. Protecting healthcare workers by pre-exposure prophylaxis.
12.	Interim Summary Guidelines for Clinical Management of Patients with COVID 19	MRI, Sri Lanka	Instructions for HCWs
13.	Community case management during an influenza outbreak: A training package for community health workers	WHO, Global	Role of the CHW during an influenza outbreak, and conducting community education activities and delivering appropriate messages specific to prevention and control of an influenza outbreak
14.	COVID-19: protecting health-care workers	The Lancet	Adequate provision of PPE is just the first step; other practical measures must be considered, including cancelling non-essential events to prioritise resources; provision of food, rest, family support; and psychological support. Presently, health-care workers are every country's most valuable resource.
15.	Protecting Health Care Workers during the COVID-19 Coronavirus Outbreak – Lessons from Taiwan's SARS response	Oxford University Press	Recommends implementing Traffic Control Bundling (TCB) – a tool that proved effective in dramatically reducing infection rates among HCWs in Taiwan during the SARS outbreak.

[1] The resource has either been deleted or moved subsequently. However, the pdf of the same is available with RES authors

			The essence of TCB involves: Triage outside of hospitals (in tents or other shelters) – ensuring patients are triaged in outdoor screening stations to ensure ill patients are directed to a contamination zone, and; Zones of Risk – clearly delineating separate zones, including a contamination, transition, and clean zone each separated by checkpoints
16.	Occupational risks for COVID-19 infection	Oxford University Press	All health personnel should be alert to the risk of COVID-19 in a wide variety of occupations, and not only HCWs. These occupational groups can be protected by good infection control practices. These at-risk groups should also be given adequate social and mental health support.
17.	Healthcare Personnel Preparedness Checklist for COVID-19	CDC	Front-line healthcare personnel in the United States should be prepared to evaluate patients for coronavirus disease 2019 (COVID-19). The document provides a checklist highlighting key steps for healthcare personnel in preparation for transport and arrival of patients with confirmed or possible COVID-19.
18.	What healthcare personnel should know about caring for patients with confirmed or possible coronavirus disease 2019 (COVID-19)	CDC	Healthcare personnel caring for patients with confirmed or possible COVID-19 should adhere to CDC recommendations for infection prevention and control (IPC)
19.	Healthcare Professionals: Frequently Asked Questions and Answers	CDC	CDC currently recommends a cautious approach to persons under investigation (PUI) for COVID-19. Healthcare personnel evaluating PUI or providing care for patients with confirmed COVID-19 should use, Standard Transmission-based Precautions.
20.	CDC Statement for Healthcare Personnel on Hand Hygiene during the Response to the International Emergence of COVID-19	CDC	CDC recommends the use of alcohol-based hand sanitizers with greater than 60% ethanol or 70% isopropanol as the preferred form of hand hygiene in healthcare settings, based upon greater access to hand sanitizer.
21.	Emergency Responders: Tips for taking care of yourself	CDC	People with pre-existing mental health conditions should continue with their treatment plans during an emergency and monitor for any new symptoms.

22.	MOH Pandemic Readiness and Response Plan for Influenza and other Acute Respiratory Diseases	Ministry of Health, Singapore	Front-line HCWs working at the restructured hospitals, polyclinics and participating primary care clinics may be given antiviral prophylaxis to protect them during the peak of the epidemic.
23.	Healthcare Workers Handbook on Influenza	National Institute for Communicable Diseases, South Africa	Page-17: IPC precautions when caring for patients with suspected, probable, or confirmed infection with influenza viruses, or ILI include precautions while working in direct contact with patients, Standard and Droplet should be applied.
24.	Guidance for Healthcare Worker Self-Monitoring and Work Restriction In the Presence of Sustained Community Transmission of Coronavirus Disease 2019 (COVID-19)	New York City Department of Health and Hygiene, USA	Page 1-2: All healthcare workers should self-monitor for illness consistent with COVID-19 because all healthcare workers are at risk for unrecognized exposures. A healthcare worker who has had a known high-risk exposure to a patient(s) with confirmed COVID-19, should take extra care to monitor his/her health but can keep working. There is no requirement for 14-day quarantine of healthcare workers with high-risk exposures in the setting of sustained community transmission.

CDC – Centres for Disease Control and Prevention, WHO – World Health Organization, MRI – Medical Research Institute, HCW- Healthcare Worker, CHW- Community Health Worker, PPE- Personal Protective Equipment, EU- European Union, EEA- European Economic Area, IPC- Infection Prevention & Control

Appendix 6 – Inventory of self-isolation guidelines in relation to COVID-19 control

No.	Document title	Issuing authority/Country of origin	Notes
1.	Information for people who are self-isolating due to contact with a case of COVID-19 or travel to an affected area	BC Centre for Disease Control, Canada	Clear instructions on self-isolation especially for inbound travellers
2.	Home isolation guidance when unwell (suspected or confirmed cases)	Department of Health, Australia	Very basic guidelines on who should be isolated ONLY in case of suspected/confirmed cases
3.	COVID-19 – Self-isolation for Close Contacts	Ministry of Health, New Zealand	Self-isolation/precaution guidelines for inbound travellers
4.	Know the Difference : Self-monitoring, self-isolation, and isolation for COVID-19	Government of Canada, Canada	Basic difference and instruction when to self-monitor/self-isolate/get isolated
5.	Patient information sheet for self-isolation at home	Health Protection Surveillance Centre, Ireland	This is detailed with clear instructions for self-isolated suspected/confirmed cases
6.	Healthcare Worker COVID-19 Close Contact Information Leaflet	Health Protection Surveillance Centre, Ireland	Quarantine order for possible COVID-19 cases because of contact
7.	Guidelines for home quarantine	MoHFW,GoI, India	Clear instructions for suspected quarantined people
8.	Interim Guidelines for Home Quarantine	Department of Health, Philippines	
9.	National Action Plan for corona virus disease (COVID-19), Pakistan	Ministry of National Health Services,Pakistan	Annexures are useful. Guidelines on self-quarantine/mitigation from a neighbour.
10.	Guideline for the Home quarantine / Quarantine in non-health care settings	Epidemiological Unit- MoH, Sri Lanka	

MOHFW- Ministry of Health & Family Welfare, GOI- Government of India, MoH- Ministry of Health

Appendix 7 – Inventory of IEC materials in relation to COVID-19 control and response produced by different organisations/authorities worldwide

No.	Title	Issuing authority/ Country of origin	Notes
**COVID-19 details-Symptoms and transmissions**			
1.	Coronavirus Disease 2019 (COVID-19) Outbreaks in Residential Care Facilities	Department of Health, Australia	Page 7-8 has good information on what is COVID and its transmission modes.
2.	COVID 19 FAQs	Department of Health, Philippines	
**Which people are at higher risk of getting infected?**			
3.	Are You at Higher Risk for Severe Illness?	CDC, USA	Advisory for high risk groups: aged, pregnant, asthma, HIV, etc.
4.	Coronavirus disease 2019 (COVID-19) Situation Report – 51	WHO, Global	Page 2: risk communication guidance for high risk group
5.	COVID-19 PUBLIC HEALTH GUIDANCE Self-Isolation for Older Adults and Those Who Have Elevated Risk	California Department of Public Health, USA	Very detailed guidance for high-risk groups
**Myth VS reality on COVID 19**			
6.	Coronavirus disease (COVID-19) advice for the public: Myth busters	WHO, Global	WHO's myth vs reality fact-check
7.	SHARE FACTS ABOUT COVID-19	CDC, USA	Few facts on COVID
8.	10 WhatsApp forwards you should not forward		Misleading social media communications
**Key Messages to spread in the community for prevention of COVID 19**			
9.	Steps to help prevent the spread of COVID-19 if you are sick	CDC, USA	Guidelines for suspected COVID
10.	Guidelines on Prevention of Coronavirus Disease 2019 (COVID-19) for the General Public	Centre for Health Protection, DoH, Hong Kong	Comprehensive guidelines: general public/inbound travellers
11.	COVID-19 CORONAVIRUS FREQUENTLY ASKED QUESTIONS	National Institute for Communicable Diseases, South Africa	Pages 2-3: protection measures for general public/inbound travellers

12.	Interim Guidelines for 2019-nCOV response in residential communities	Department of Health, Philippines	From a developing country
13.	Novel Coronavirus (2019-nCoV)-General Advisory for Public	Dept. of Health & Family Welfare, Delhi Government	General advisory for public (very generic)
**For those showing symptoms or have travelled to other countries or states in past 2 weeks**			
14.	Travelers from Countries with Widespread Sustained (Ongoing) Transmission Arriving in the United States	CDC, USA	Very broad brief guidelines for inbound travellers
15.	Instructions for Self-Isolation for Iowans Returning from Countries Affected by COVID-19	IOWA Department of Public Health, USA	Self-isolation guidelines for inbound travellers from COVID countries
16.	Advices for Sri Lankan Students/Nationals and other foreign nationals returning from high risk area of on-going Corona virus transmission	Epidemiological Unit MoH, Sri Lanka	
17.	***10 and 11 is relevant too***		
**How to take care of yourself (FLHWs) and carry on with your other duties?**			
18.	***On Appendix 5***		

DoH- Department of Health
